# Serum Irisin Level Is Positively Associated with Bone Mineral Density in Patients on Maintenance Hemodialysis

**DOI:** 10.1155/2021/8890042

**Published:** 2021-01-25

**Authors:** Chia-Wen Lu, Chih-Hsien Wang, Yu-Li Lin, Chiu-Huang Kuo, Yu-Hsien Lai, Bang-Gee Hsu, Jen-Pi Tsai

**Affiliations:** ^1^Division of Nephrology, Hualien Tzu Chi Hospital, Buddhist Tzu Chi Medical Foundation, Hualien, Taiwan; ^2^Institute of Medical Sciences, Tzu Chi University, Hualien, Taiwan; ^3^School of Medicine, Tzu Chi University, Hualien, Taiwan; ^4^School of Post-Baccalaureate Chinese Medicine, Tzu Chi University, Hualien, Taiwan; ^5^Division of Nephrology, Department of Internal Medicine, Dalin Tzu Chi Hospital, Buddhist Tzu Chi Medical Foundation, Chiayi, Taiwan

## Abstract

**Background:**

Irisin is a circulating hormone-like myokine that plays an important role in bone metabolism. We performed a cross-sectional study to investigate whether serum irisin levels correlated with bone mineral density (BMD) in patients on maintenance hemodialysis (MHD).

**Methods:**

Blood samples were obtained from 80 patients on MHD, and serum irisin concentrations were determined using a commercially available enzyme-linked immunosorbent assay. BMD was measured by dual-energy X-ray absorptiometry of the L2–L4 vertebrae.

**Results:**

In the study cohort, 10 (12.5%) and 19 (23.8%) patients had osteoporosis and osteopenia, respectively, and 51 (63.75%) patients had normal BMD. Lumbar T-score was negatively associated with body height (*P*=0.010), body weight (*P*=0.002), body mass index (BMI, *P*=0.010), and serum irisin (*P* < 0.001) and was positively associated with advanced age (*P*=0.031), female sex (*P*=0.001), alkaline phosphatase (ALP, *P*=0.010), urea reduction rate (*P*=0.018), and fractional clearance index for urea (*P*=0.020). Multivariable forward stepwise linear regression analysis revealed that high serum logarithmically transformed irisin (log-irisin, *β* = 0.450, adjusted *R*^2^ change = 0.258; *P* < 0.001), female sex (*β* = −0.353, adjusted *R*^2^ change = 0.134; *P* < 0.001), and serum ALP level (*β* = −0.176, adjusted *R*^2^ change = 0.022; *P*=0.049) were significantly and independently associated with lumbar BMD in patients on MHD.

**Conclusions:**

In addition to female sex and serum ALP level, serum irisin level was positively associated with lumbar BMD in patients on MHD.

## 1. Introduction

It was well known that mineral and bone disorders are associated with increased fracture risk and substantial morbidity and mortality in patients with chronic kidney disease (CKD) compared with the general population [1–3]. Osteoporosis, characterized by low bone mass and density, is associated with an increased fracture risk and significant public health burden globally in the general population as well as in patients with end-stage renal disease (ESRD) [3, 4]. In Taiwan, an epidemiological study of the general population aged above 50 years reported that the prevalence of osteoporosis increased from 17.4% in 2001 to 25% in 2011 [5]. Abnormal bone turnover is common and gradually declines with worsening renal function in patients with CKD. Indeed, the 2017 Kidney Disease: Improving Global Outcomes (KDIGO) guidelines recommend bone mineral density (BMD) examination by dual-energy X-ray to assess fracture risk in patients with CKD based on studies showing that decreased BMD is common in patients on maintenance hemodialysis (MHD), with osteoporosis and osteopenia rates of 9.5%–23% and 16.7%–45%, respectively [6–8].

To elucidate the increased predisposition of patients with CKD and low BMD to osteoporosis and fractures, several studies investigated factors associated with BMD including age, sex, nutrition, physical activity, body composition, and myokine levels [9,10]. Evidence showed that inflammation could influence the mechanism of osteoclastogenesis as well as bone resorption in late-stage CKD and MHD patients [11]. Irisin, a newly discovered myokine, which is cleaved from the precursor fibronectin type III domain-containing 5 (FNDC5), is primarily known for its modulatory role in phenotypic changes of white adipose tissue to brown adipose tissue to increase energy expenditure and improve glucose homeostasis; irisin also activates promyogenic genes for subsequent activation of satellite cells and increased protein synthesis [12, 13]. Based on its increased production and release by myocytes after exercise, irisin is considered as an anabolic mediator between the muscle and bone both *in vitro* and *in vivo* [14, 15]. In athletes and healthy individuals, irisin positively correlates with total body and hip BMD and strength [16, 17]. However, irisin has also been reported to exhibit a negative relationship with osteoporosis and fracture independently of BMD, body composition, and physical activity in postmenopausal women [18, 19]. A meta-analysis showed that serum irisin was decreased in elderly women with osteoporosis and exhibited a positive correlation with BMD [9].

Overall, these studies highlight the potential role of irisin as a marker and modulator of abnormalities in muscle and bone, but its role on bone density in MHD patients remains unclear. Therefore, we evaluated risk factors for osteoporosis and the relationship between serum irisin levels and BMD in patients on MHD.

## 2. Materials and Methods

### 2.1. Patients

This cross-sectional study conducted at Hualien Tzu Chi Hospital, a medical center in Hualien, Taiwan, between June 2015 and August 2015 included patients above 50 years of age on MHD using the standard 4-hour hemodialysis three times a week with standard bicarbonate dialysate and high-flux polysulfone disposable artificial kidney (FX class dialyzer, Fresenius Medical Care, Bad Homburg, Germany). Patients fulfilling the following criteria were excluded: treatment with antiosteoporotic medication (bisphosphonates, teriparatide, or estrogen medications), history of lumbar fracture or surgery, acute infection, malignancy, acute myocardial infarction, pulmonary edema, heart failure at the time of blood sampling, and refusal to provide informed consent. A total of 80 patients fulfilling these criteria were included in the final analyses. The study was approved by the Research Ethics Committee of Hualien Tzu Chi Hospital, Buddhist Tzu Chi Medical Foundation (IRB106-62-B), and is conducted in accordance with the World Medical Association Declaration of Helsinki.

### 2.2. Anthropometric Analysis

Body weight and height were measured to the nearest half kilogram and half centimeter, respectively, with patients in light clothing and without shoes. Body mass index (BMI) was calculated as body weight (kg) divided by body height squared (m^2^) [20–22].

### 2.3. Biochemical Analyses

Fasting blood samples (approximately 5 mL) were immediately centrifuged at 3000 g for 10 minutes after collection before the hemodialysis. Serum samples were stored at 4°C. Biochemical analyses were performed within one hour of collection. Serum levels of blood urea nitrogen, creatinine, alkaline phosphatase (ALP), total calcium, and phosphorus were measured using an autoanalyzer (Siemens Advia 1800, Siemens Healthcare, Henkestr, Germany). Fractional clearance index for urea (Kt/V) and urea reduction ratio (URR) were measured before and immediately after hemodialysis using a formal, single-compartment dialysis urea kinetic model. Commercial enzyme-linked immunosorbent assays were used to measure serum levels of irisin (Phoenix Pharmaceuticals, CA, USA; catalogue number: EK-067-29) and intact parathyroid hormone (PTH) (IBL International GmbH, Hamburg, German; catalogue number: NM59041) [22]. The interassay and intraassay coefficient of variation of irisin is less than 15% and 10% and interassay and the intraassay coefficient of variation of intact PTH is 5.7% and 4.8%, respectively.

### 2.4. Measurement of Bone Mineral Density

BMD was measured immediately after blood sampling before MHD. The BMD of L2–L4 vertebrae was measured using dual-energy X-ray absorptiometry (QDR 4500, Hologic, MA, USA) and expressed using absolute values (g/cm^2^) and T-scores (deviation from peak BMD) [20–22]. T-score was defined as the number of standard deviations from the mean BMD of sex-matched young control subjects. Compared to the mean BMD of controls, a lumbar bone T-score less than −2.5 was used as the diagnostic cutoff for osteoporosis and a lumbar bone T-score between −1.0 and −2.5 was used for the diagnosis of osteopenia, according to the World Health Organization criteria [23].

### 2.5. Statistical Analysis

Data were tested for normal distribution using the Kolmogorov–Smirnov test. Data were expressed as means ± standard deviation for normally distributed data and as medians with interquartile ranges for nonnormally distributed data. Categorical variables were analyzed using the *χ*^2^ test. The significance of differences among the normal, osteopenia, and osteoporosis groups was determined using the Kruskal–Wallis or one-way analysis of variance, based on the normality of data and Dunn's multiple comparison test or post hoc Bonferroni test for multiple comparisons. The duration of hemodialysis and the levels of albumin and irisin exhibited skewed distributions; therefore, the data were log-transformed to achieve normality. Clinical variables that correlated with lumbar BMD in patients on MHD were evaluated by simple linear regression analysis and multivariable forward stepwise linear regression analysis. All statistical analyses were performed using SPSS for Windows (version 19.0; SPSS, Chicago, IL, USA). A *P* value of <0.05 was considered to indicate statistical significance.

## 3. Results

Based on BMD measurements, the study cohort comprised 51 (63.75%), 19 (23.75%), and 10 (12.5%) patients in the normal, osteopenia, and osteoporosis groups, respectively (Table 1). Compared with the normal group, the rates of female and older patients were significantly higher in the osteopenia and osteoporosis groups (*P*=0.001 and *P*=0.031, respectively). Additionally, the mean BMI and mean serum irisin levels were lower (*P*=0.010 and *P* < 0.001, resp.) and the mean ALP, URR, and Kt/V (*P*=0.01, *P*=0.018, and *P*=0.02, respectively) were higher in the osteopenia and osteoporosis groups compared with the normal group. The rates of diabetes mellitus and hypertension as comorbidities did not differ among the three groups.

By simple linear regression analysis, body height (*r* = 0.440, *P* < 0.001), body weight (*r* = 0.462, *P* < 0.001), BMI (*r* = 0.332, *P* < 0.003), serum creatinine (*r* = 0.297, *P* < 0.007), and log-transformed irisin level (log-irisin, *r* = 0.517, *P* < 0.001) were positively correlated with lumbar BMD whereas female sex (*r* = −0.430, *P* < 0.001), age (*r* = −0.268, *P*=0.016), serum ALP level (*r* = −0.294, *P*=0.008), URR (*r* = −0.271, *P*=0.015), and Kt/V (*r* = −0.268, *P*=0.016) were negatively correlated with lumbar BMD (Table 2). The multivariable forward stepwise linear regression analysis including variables that were associated with lumbar BMD in the simple linear regression analysis (sex, age, BMI, creatinine, ALP, URR, Kt/V, and log-irisin) revealed that serum log-irisin level (*β* = 0.450, adjusted *R*^2^ change = 0.258; *P* < 0.001), female sex (*β* = −0.353, adjusted *R*^2^ change = 0.134; *P* < 0.001), and serum ALP level (*β* = −0.176, adjusted *R*^2^ change = 0.022; *P* < 0.049) were significantly and independently associated with lumbar BMD in patients on MHD.

## 4. Discussion

The present cross-sectional analysis of 80 patients on MHD revealed that the serum irisin levels were lower in patients with osteopenia or osteoporosis compared to those with normal BMD. Furthermore, in addition to female sex, serum ALP level was negatively correlated, while serum irisin level was positively correlated lumbar BMD in patients on MHD.

Several studies aimed to identify risk factors for low BMD in patients with CKD or ESRD who are at a higher risk of osteoporosis and fracture-related mortality compared to the general population [1, 10, 24, 25]. In a cohort study of patients on CKD not undergoing dialysis, Hyun et al. showed that low BMD was prevalent, with osteopenia and osteoporosis rates of 33% and 8%, respectively; the authors also reported that low BMD was significantly associated with female sex, old age, low BMI, and decreased renal function as well as increased risk of ESRD [26]. In another longitudinal cohort study, Nickolas et al. found that patients on CKD experienced progressive loss of cortical density and thickness along with increased porosity in the bone, which were driven by increased bone turnover based on PTH and bone-specific ALP levels; the authors also found that PTH and bone-specific ALP levels predicted the decreases in the cortical area (2.2% and 2.8%, respectively.) and cortical thickness (2.0% and 2.5%, respectively.) [27]. Moreover, another study utilizing dual-energy X-ray absorptiometry reported that 13.6%, 22.2%, and 33.3% of patients on dialysis exhibited osteoporosis of the spine, hip, and any site (hip and spine), respectively [28]. In addition, the authors found that BMD was negatively associated with age, female sex, and bone-specific ALP level and positively associated with BMI; however, BMD did not show significant associations with dialysis duration, diabetes mellitus, and smoking [28]. In agreement with these studies, we also found that 23.75% and 12.5% of the patients on MHD had osteopenia and osteoporosis, respectively, and that MBD was negatively associated with age, female sex, and ALP and positively associated with BMI.

Physical exercise is crucial for the healthy development of the skeleton, whereas muscle disuse in patients with sarcopenia can lead to osteoporotic hip fractures, which are associated with reduced life expectancy and increased mortality [29]. Serum levels of irisin, a myokine cleaved from FNDC5, increases after exercise, indicating the link between muscle and bone activity [30]. Exercise was shown to induce irisin expression compared to the resting state in mice, and conditioned medium from exercised myoblasts was demonstrated to target osteoblasts and enhance the differentiation of bone marrow stromal cells, indicating the osteogenic effect of muscle [14]. Moreover, injection of recombinant irisin at a lower dose before the induction of browning in white adipose tissue of mice was associated with improved cortical mineral density, bone bending strength, and geometrical architecture as well as with a reduction in osteoclasts [15]. In mice subjected to mechanical unloading, recombinant irisin induced the preservation of cortical and trabecular BMD and bone volume by reducing sclerostin and increasing osteoprotegerin to the levels measured in normally ambulated mice and by restoring osteoblastogenesis [31]. Recently, Zhu et al. reported that the novel *Fndc5*/irisin knockout mice exhibited lower BMD, bone surface, and bone volume, with delayed bone development and hypomineralization [32]. The authors also showed that the treatment of bone marrow-derived mesenchymal cells with recombinant irisin induced osteoblastogenesis through the activation of Wnt/*β*-catenin pathway and inhibited osteoclastogenesis through the inhibition of receptor activator of nuclear factor-*κ*B ligand-induced AKT cascade and suppression of NFATc1 activation. Altogether, these findings indicate that exercise-induced release of irisin has profound effects on bone remodeling as an anabolic mediator between muscle and bone. In clinical studies, irisin was shown to be a stronger determinant of bone mineral status compared with PTH and bone ALP in children [33]. In athletes and older individuals, serum irisin levels were positively correlated with total body and subregional anatomical BMD [16, 17]. Furthermore, irisin was reported to be negatively correlated with osteoporosis and fracture independently of BMD, body composition, and physical activity in postmenopausal women [18, 19]. In a meta-analysis, there was a weak association between irisin and lumbar BMD as well as femoral neck fracture; decreased irisin levels were associated with osteoporosis by pooled analysis and irisin levels were even lower in postmenopausal women and those with a history of fracture [9], suggesting that irisin might have utility as a marker for the assessment of metabolic bone disease. However, Kim et al. found irisin could induce the expression of sclerostin in vitro in a dose-dependent manner, which indicated suppression of the activity of osteoblast [34]. Moreover, FNDC5 knockout mice displayed suppressed RANKL activity, which was correlated with the reduction of bone resorption [34]. According to the consideration by Kim et al., irisin could be a molecule similar to parathyroid home which could exert both beneficial and harmful effects through mechanisms such as constant high level or intermittent pulse levels on bone health. Taken together, we found that there was a positive association between lumbar BMD and serum irisin level independently of baseline comorbidities, dialysis duration, female sex, and age. These findings highlight the potential role of irisin as a modulator and marker of bone health in patients on MHD. But the mechanism of irisin on bone health in MHD should be further studied to delineate the actual role of irisin as a mediator of negative feedback control or a marker of decreased muscle mass as well as impaired physical activities in this frail population.

One major limitation of the present study was the cross-sectional design and the inclusion of a limited number of patients on MHD. In addition, evidence showed daily activities would influence the serum levels of irisin but there were no available data on the daily activity of patients. Therefore, the causal relationship between irisin and BMD and the underlying mechanisms should be confirmed by future longitudinal studies.

In the present study, lumbar BMD was positively correlated with serum irisin level in addition to female sex and serum ALP level in patients on MHD. These findings indicate that irisin might serve as an endocrine signal in the pathogenesis of osteoporosis and might be considered as a novel therapeutic target to restore bone mass in osteoporosis caused by muscle disuse in certain chronic diseases. Further, our findings suggest that irisin should be considered as a useful biomarker for osteoporosis in patients on MHD, which requires further confirmation in specifically designed studies.

## Figures and Tables

**Table 1:** Clinical characteristics according to different lumbar T-score cutoff points (normal, osteopenia, and osteoporosis) of the 80 hemodialysis patients.

Characteristics	All patients (*n* = 80)	Normal (*n* = 51)	Osteopenia (*n* = 19)	Osteoporosis (*n* = 10)	*P*
Age (years)	66.93 ± 10.27	64.71 ± 10.10	70.11 ± 9.95	72.20 ± 9.00	0.031^*∗*^
Hemodialysis duration (months)	57.66 (23.82–122.34)	56.88 (19.44–114.96)	81.00 (34.44–145.20)	47.58 (15.72–164.61)	0.484
Height (cm)	159.74 ± 8.28	161.80 ± 7.80	156.47 ± 7.18^†^	155.40 ± 9.63	0.010^*∗*^
Body weight (kg)	64.13 ± 14.55	67.95 ± 14.32	60.41 ± 10.13	51.75 ± 15.15^‡^	0.002^*∗*^
Body mass index (kg/m^2^)	24.99 ± 4.66	25.88 ± 4.78	24.64 ± 3.76	21.12 ± 3.77^‡^	0.010^*∗*^
Lumbar bone mineral density (g/cm^2^)	0.95 ± 0.20	1.07 ± 0.14	0.80 ± 0.06^†^	0.65 ± 0.05^‡,a^	<0.001^*∗*^
Lumbar T-score	−0.51 ± 1.60	0.45 ± 1.10	−1.73 ± 0.44^†^	−3.03 ± 0.38^‡,a^	<0.001^*∗*^
Lumbar Z-score	0.57 ± 1.06	1.08 ± 0.76	0.08 ± 0.61^†^	−1.09 ± 0.87^‡,a^	<0.001^*∗*^
Systolic blood pressure (mmHg)	136.60 ± 24.37	140.72 ± 24.56	132.16 ± 21.27	124.00 ± 25.36	0.091
Diastolic blood pressure (mmHg)	73.23 ± 13.78	73.35 ± 15.10	71.16 ± 9.67	66.30 ± 11.14	0.124
Albumin (mg/dL)	4.10 (3.90–4.40)	4.10 (3.90–4.40)	4.10 (4.00–4.10)	4.20 (3.68–5.25)	0.630
Blood urea nitrogen (mg/dL)	58.74 ± 14.47	57.71 ± 13.65	61.00 ± 10.11	59.70 ± 16.30	0.687
Creatinine (mg/dL)	9.22 ± 1.79	9.49 ± 1.81	8.78 ± 1.67	8.65 ± 1.98	0.186
Alkaline phosphatase (U/L)	83.86 ± 29.95	78.65 ± 27.50	84.42 ± 29.87	109.40 ± 31.74^‡^	0.010^*∗*^
Total calcium (mg/dL)	8.96 ± 0.78	8.91 ± 0.77	9.15 ± 0.82	8.96 ± 0.78	0.464
Phosphorus (mg/dL)	4.62 ± 1.21	4.71 ± 1.13	4.67 ± 1.25	4.07 ± 1.54	0.310
Intact parathyroid hormone (pg/mL)	225.72 ± 182.18	208.59 ± 178.27	266.92 ± 185.17	234.84 ± 202.18	0.491
Irisin (ng/mL)	39.99 (12.51–105.84)	54.92 (17.46–161.30)	34.19 (12.99–56.16)^†^	3.01 (1.52–9.03)^‡,a^	<0.001^*∗*^
Urea reduction rate	0.73 ± 0.04	0.72 ± 0.04	0.75 ± 0.04^†^	0.75 ± 0.04	0.018^*∗*^
Kt/V (Gotch)	1.33 ± 0.17	1.29 ± 0.16	1.40 ± 0.16	1.41 ± 0.18	0.020^*∗*^
Female, *n* (%)	39 (48.8)	17 (33.3)	15 (78.9)	7 (70.0)	0.001^*∗*^
Diabetes mellitus, *n* (%)	33 (41.3)	25 (49.0)	6 (31.6)	2 (20.0)	0.145
Hypertension, *n* (%)	35 (43.8)	24 (47.1)	7 (36.8)	4 (40.0)	0.722

Values for continuous variables given as means ± standard deviation and test by one-way analysis of variance; variables not normally distributed given as medians and interquartile range and test by Kruskal–Wallis analysis. ^*∗*^*P* < 0.05 was considered statistically significant after the Kruskal–Wallis analysis or one-way analysis of variance.^†^Compared with the normal group and osteopenia group, ^‡^compared with normal group and osteoporosis group, and ^a^compared with osteopenia group and osteoporosis group was and <0.05 considered statistically significant after Dunn's multiple comparison test or post hoc Bonferroni test. Kt/V, fractional clearance index for urea.

**Table 2:** Correlation of lumbar BMD levels and clinical variables by simple regression or multivariable linear regression analyses among the 80 hemodialysis patients.

Variables	Lumbar BMD (g/cm^2^)				
	Simple regression		Multivariable regression		
	*r*	*P*	Beta	Adjusted *R*^2^ change	*P*
Female	−0.430	<0.001^*∗*^	−0.353	0.134	<0.001^^*∗*^^
Diabetes mellitus	0.164	0.146	—	—	—
Hypertension	0.162	0.152	—	—	—
Age (years)	−0.268	0.016^*∗*^	—	—	—
Log-HD duration (months)	−0.054	0.631	—	—	—
Body mass index (kg/m^2^)	0.332	0.003^*∗*^	—	—	—
Systolic blood pressure (mmHg)	0.208	0.064	—	—	—
Diastolic blood pressure (mmHg)	0.200	0.075	—	—	—
Log-albumin (mg/dL)	0.022	0,848	—	—	—
Blood urea nitrogen (mg/dL)	0.035	0.757	—	—	—
Creatinine (mg/dL)	0.297	0.007^*∗*^	—	—	—
Alkaline phosphatase (U/L)	−0.294	0.008^*∗*^	−0.176	0.022	0.049^*∗*^
Total calcium (mg/dL)	−0.022	0.849	—	—	—
Phosphorus (mg/dL)	0.149	0.188	—	—	—
Intact parathyroid hormone (pg/mL)	−0.137	0.244	—	—	—
Log-irisin (ng/mL)	0.517	<0.001^*∗*^	0.450	0.258	<0.001^*∗*^
Urea reduction rate	−0.271	0.015^*∗*^	—	—	—
Kt/V (Gotch)	−0.268	0.016^*∗*^	—	—	—

Data of HD duration, albumin, and irisin showed skewed distribution and therefore were log-transformed before analysis. Analysis of data was done using the univariate linear regression analyses or multivariate stepwise linear regression analysis (adapted factors were female, age, body mass index, creatinine, alkaline phosphatase, urea reduction rate, Kt/V, and log-irisin). HD, hemodialysis; Kt/V, fractional clearance index for urea. ^*∗*^*P* < 0.05 was considered statistically significant.

## Data Availability

The datasets used and analyzed during the current study are available from the corresponding author on reasonable request.
